# The Association of miRNA10a and Glucose Transporters in Oral Squamous Cell Carcinoma With Diabetes: A Pilot Study

**DOI:** 10.7759/cureus.51752

**Published:** 2024-01-06

**Authors:** Sukanth R, Priyadharshini R, Selvaraj Jayaraman, Sinduja Palati

**Affiliations:** 1 Department of General Pathology, Saveetha Dental College and Hospitals, Saveetha Institute of Medical and Technical Sciences, Saveetha University, Chennai, IND; 2 Department of Oral and Maxillofacial Pathology, Saveetha Dental College and Hospitals, Saveetha Institute of Medical and Technical Sciences, Saveetha University, Chennai, IND; 3 Centre of Molecular Medicine and Diagnostics, Department of Biochemistry, Saveetha Dental College and Hospitals, Saveetha Institute of Medical and Technical Sciences, Saveetha University, Chennai, IND

**Keywords:** expression, squamous cell carcinoma, mirna, innovative, glut

## Abstract

Introduction: MicroRNAs (miRNAs) are well-established post-translational non-coding RNAs that play crucial roles in mRNA degradation and repression. Glucose transporter 1 (GLUT1) showed correlation along with various miRNA, specifically miRNA10a expression in lung cancers. The role of miRNA10a along with glucose upregulation leading to cancer proliferation in oral squamous cell carcinoma (OSCC) is unknown. This study aimed to investigate the expression levels of miRNA10a and GLUT1 in OSCC patients with diabetes.

Materials and methods: miRNA10a and GLUT1 expression were estimated in OSCC, precancerous, and healthy tissues using quantitative reverse transcriptase polymerase chain reaction (RT-PCR). miRNA10a and GLUT1 expression levels were recorded as fold change. Further, a one-way analysis of variance (ANOVA) test was performed to find whether there is any difference in miRNA10a and GLUT1 expression between OSCC, precancerous, and healthy tissues.

Results: The RT-PCR findings revealed an increased expression of miRNA10a and GLUT1 in OSCC compared to precancerous and healthy tissue. There is a positive correlation between miRNA10a and GLUT1 expression levels in both potentially malignant and control tissues, with a marked increase in cancerous tissue. This study demonstrated the significance of upregulated miRNA10a expression, indicating a direct correlation with OSCC proliferation via GLUT1 overexpression. Specifically, miRNA10a exhibited a fold change of 1.2±0.072 in potentially malignant tissue and 1.4±0.05 in cancer tissue, while GLUT1 exhibited a fold change of 1.25±0.092 in potentially malignant tissue and 0.092±0.08 in cancer tissue, respectively.

Conclusion: This research highlights the role of miRNA10a in cancer progression by facilitating proliferation through the regulation of GLUT1 in cancerous tissues, particularly in hyperglycemic conditions. This mechanism further contributes to increased glucose transport in cancer patients, which may potentially impede tumor prognosis. These findings underscore the potential significance of targeting miRNA10a and GLUT1 as therapeutic interventions in cancer management.

## Introduction

Head and neck cancer (HNC) represents a significant global health concern, with an estimated 888,000 new cases and 453,000 fatalities in 2018. Among these cases, oral squamous cell carcinoma (OSCC) stands out as the seventh most prevalent cancer worldwide, ranking fifth among males and twelfth among females. OSCC's development is intricately linked to risk factors such as smoking, alcohol consumption, poor dietary habits, and human papillomavirus (HPV) viral infections. Despite advances in therapeutic options, OSCC's prognosis remains unfavorable and has shown limited improvement over the past two decades [1]. Understanding the molecular underpinnings of OSCC's pathogenesis is a challenging task, complicating the development of effective treatment strategies [2].

In OSCC, the dysregulated molecular metabolic pathways represent a focal point of investigation. These pathways predominantly hinge on aerobic glycolysis, for adenosine triphosphate (ATP) production. Within this metabolic landscape, glucose transporter 1 (GLUT1) emerges as a pivotal protein, governing the facilitation of glucose transportation into mammalian cells. This aberrant upsurge in energy production mechanisms within tumor cells has sparked significant interest in targeting and inhibiting these metabolic pathways as a potential avenue for cancer therapy [3]. Recent research has highlighted the promise of miRNAs as ideal biomarkers for early cancer diagnosis and prognosis. miRNAs, a class of short non-coding RNAs, exert their influence by targeting specific miRNAs, thereby modulating a wide array of physiological processes. Their stability in human peripheral blood and bodily fluids, coupled with disease-specific expression patterns, makes them attractive candidates for cancer-related investigations [4].

miRNA10a has been identified as an oncogenic miRNA in lung and oral cancer, driving cancer development and progression through its interaction with the phosphatase and tensin homolog signaling pathway thereby promoting cancer progression by altering the glucose metabolism which promotes cancer cell migration and adhesion [5,6]. Aberrant upregulation of GLUT1 has been documented in various human cancers, contributing to cancer cell proliferation through increased glucose metabolism [7,8]. Within the realm of cancer biology, microRNAs (miRNAs) have emerged as crucial regulators with the potential to affect energy metabolism, particularly through the regulation of GLUT1 expression [9]. miRNA10a showed an oncogenic role in the progression of lung and oral cancer [5,10]. However, many studies haven't analyzed the correlation of miRNA10a and GLUT1 expression. This study aimed to analyze the association of miRNA10a and GLUT1 in OSCC in diabetic patients.

## Materials and methods

Participant selection

Participants were selected from the oral oncology centre. Ethical approval for this study was obtained from the Scientific Research Committee prior to patient recruitment (IHEC/SDC/FACULTY/22/GPATH/440). Informed consent was taken from all the participants. OSCC patients were selected based on the following eligibility criteria.

Inclusion Criteria

(1) Individuals included in the study were aged 40 years or older, diagnosed with type 2 diabetes, had blood sugar levels exceeding 200 mg/dl, and were currently undergoing excisional biopsy for OSCC. (2) Patients who were under continuous medical supervision, and their medical records were available for review. (3) Patients with no previous history of malignancy were considered eligible for inclusion.

Exclusion Criteria

(1) Patients previously diagnosed with multiple diseases were excluded from the study. (2) Patients who had previously received treatments for malignancies were not included. (3) Samples from patients with recurrent cases of OSCC were excluded. The pilot study enrolled a total of three male and two female participants, whose ages ranged from 40 to 65 years, with a mean age of 45.3±4.4 years.

Sample collection

Cancerous and precancerous tissue samples were collected from OSCC with type 2 diabetes who underwent surgery at the centre. Healthy gingival tissue samples were collected from extracted third molars. Participants were categorized into three distinct groups as control, precancer, and cancer (n=5).

Isolation of miRNA from tissue samples

Isolation of miRNA was followed as mentioned in previous research [11]. To extract miRNA from tissue samples, we employed a Total RNA Isolation Reagent kit. Fresh tumor tissue samples (100mg) were homogenized in an RNA reagent and collected in a microfuge tube. The homogenization process was conducted at -80°C for 60 minutes and the mixture was combined with chloroform (0.2ml) and vortexed for a minute. The tissue was kept at five minutes at 4°C. Centrifugation was subjected at 4°C to 12,000 x g. Extracted samples containing miRNA were carefully transferred to a new microfuge, followed by an isopropanol wash. The samples were frozen at 4°C for 10 minutes, proceeded with centrifugation at 12,000 x g, and the pellets were washed with 75% ethanol, and centrifugated at 7,500 x g (4°C for five minutes). After discarding supernatant pellets were kept for 10 minutes at 60°C in a water bath after dissolving in 50 μl of autoclaved Milli-Q® water (Merck KGaA, Darmstadt, Germany).

RNA quantification

For RNA quantification, we employed spectrophotometry at 260/280 nm to measure absorbance in diluted RNA samples. An absorbance reading at 260 nm corresponds to 40 ng of RNA per 1 ml. To calculate the RNA quantity in the sample, we multiplied the A260 value by 40, taking into account the dilution factor. The purity of the sample was evaluated at an absorbance ratio between 260 nm to 280nm.

Principle of real-time quantitative polymerase chain reaction (PCR)

PCR was employed to amplify a target gene, generating a large number of copies. This process encompasses three primary stages: Denaturation at 94°C for three minutes, which involves melting of double-stranded DNA into single strands. Annealing was conducted at temperatures ranging from 54 to 65°C for 30 seconds, this stage establishes and disrupts ionic bonds between single-stranded template and primer. Finally, extension occurs for 30 seconds at 72°C, this stage involves the polymerase adding complementary bases to the template, resulting in the exponential amplification of the gene of interest.

Reagents

The following reagents and materials were utilized:

(1) 2X Reaction Buffer: PCR master mix kit (Takara Bio Inc., Japan), which included TaKaRa Ex Taq HS (a heat-resistant PCR enzyme), dNTP Mixture, Mg2+, Tli RNase H (minimizes PCR inhibition), SYBR Green I;

(2) Forward Primer (10μM);

(3) Reverse Primer (10μM);

(4) cDNA-Template;

(5) Autoclaved Milli-Q water;

(6) Gene-specific oligonucleotide primers were employed in the study.

Procedure

The CFX96 Real-Time PCR system (Bio-Rad, USA) was employed for real-time PCR analysis. A reaction mix of 10 μl was prepared by combining cDNA (1 μl) with sterile water (5 μl), 1 μl of forward and reverse primers (for β-actin, G-6-Pase, and PEPCK), and 0.1 μl of SYBR Green I. The protocol included denaturation for three minutes at 95°C, followed by PCR for 40 cycles. Then samples were denatured at 95°C for 10 seconds, followed by 20 seconds of annealing at 60°C and 20 seconds of extension at 72°C. Negative controls (no template controls, NTC) were included, and each reaction was run in triplicate. Amplicons in multiples were checked using melt curve analysis using thermal cycling for each sample which ranged from 50 to 95°C. The level of miRNA expression was analyzed using the CFX96 Real-Time PCR system and its associated software.

Statistical analysis

The statistical analysis in this study was conducted using one-way ANOVA through the Statistical Package for the Social Sciences (IBM SPSS Statistics for Windows, IBM Corp., Version 23.0, Armonk, NY). Results were recorded as mean ± S.E.M (standard error of the mean).

## Results

**Figure 1:**
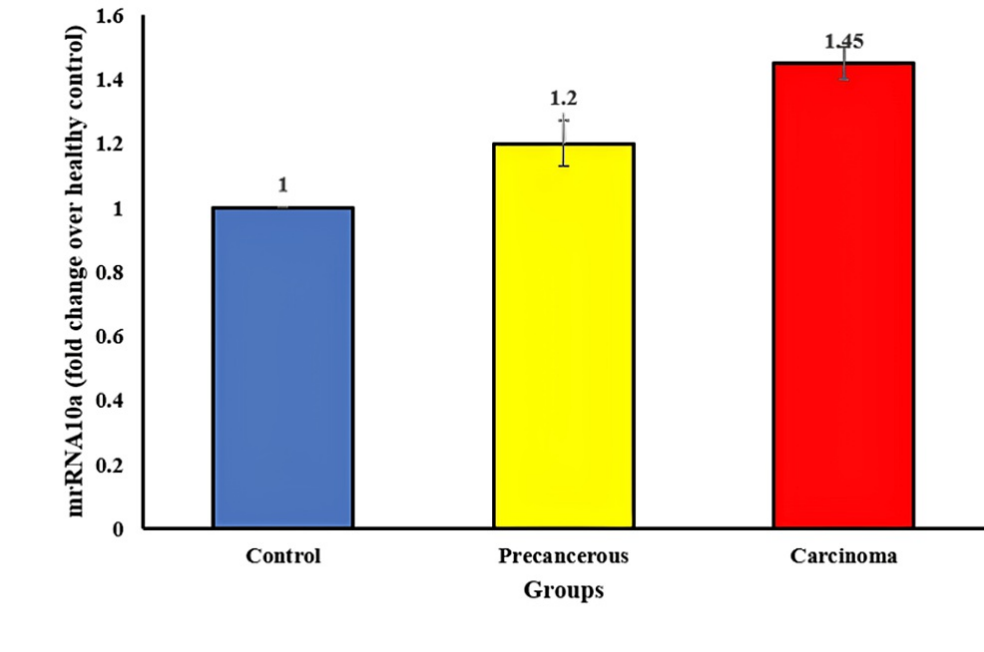
The graph exhibits an increase in miRNA10a fold change of OSCC compared to precancerous tissue and healthy control. OSCC: oral squamous cell carcinoma

Figure 1 depicts a significant increase in the fold change of miRNA10a expression in cancerous tissue compared to healthy controls. The mean fold change ± S.E.M is reported as 1.4±0.05 for the cancerous tissue samples, signifying a 1.4-fold elevation in miRNA10a expression on average in cancerous tissues compared to healthy controls. In parallel, potentially malignant tissue also displays an increased but slightly lower miRNA10a expression level, with a mean±S.E.M of 1.2±0.072. This suggests a notable elevation in miRNA10a even in these pre-cancerous tissues, albeit to a lesser extent than in fully developed cancerous tissues.

PCR data analysis highlights an exponential rise in amplification cycles ranging from 15 to 20 cycles specifically for miRNA10a. This exponential amplification signifies the substantial presence and rapid replication of miRNA10a molecules within the tissue samples. The detection of DNA formation occurring at 320 Relative Fluorescence Units (RFU) for miRNA10a further emphasizes the abundance and significance of this particular microRNA.

**Figure 2:**
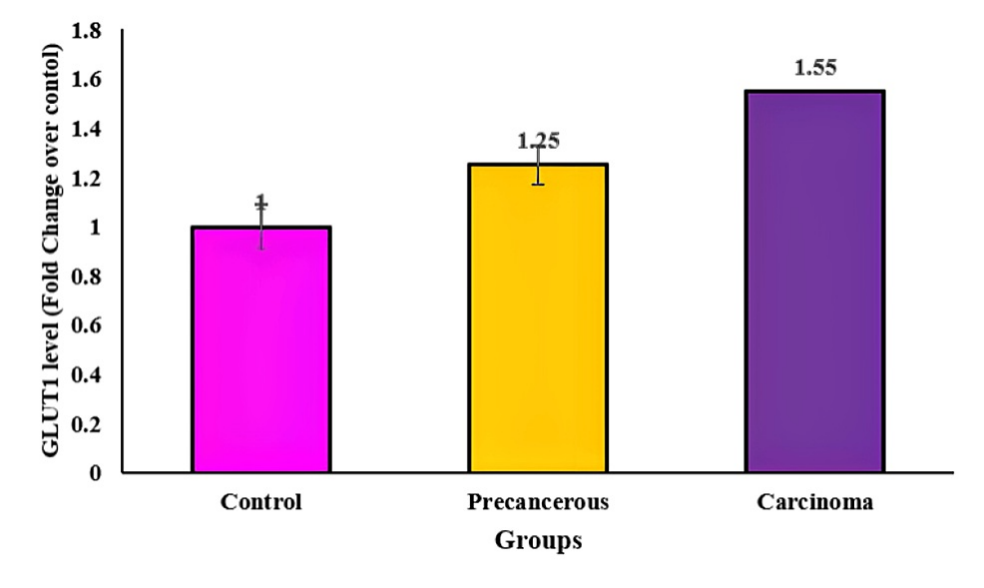
The graph exhibits an increase in GLUT1 fold change of OSCC compared to precancerous tissue and healthy control. OSCC: oral squamous cell carcinoma, GLUT1: glucose transporter 1

Conversely, Figure 2 showcases an increase in GLUT1 levels in cancerous tissue when compared to healthy control groups. The mean fold change ± S.E.M in potentially malignant tissue is reported as 1.25±0.092, indicating an elevated expression of GLUT1 even in these potentially problematic tissues. However, in fully developed cancerous tissue, the levels of GLUT1 exhibit a more substantial increase, with a mean±S.E.M of 1.15±0.08, suggesting a higher degree of upregulation compared to healthy controls.

The PCR data for GLUT1 reveals a different pattern, with amplification cycles occurring around 30 to 40 cycles. This prolonged cycle range indicates a slower but significant amplification process for GLUT1 compared to miRNA10a. The detection of DNA formation at 330 RFU specifically for GLUT1 highlights the threshold at which the amplification process successfully generates DNA copies corresponding to the presence of GLUT1.

Combining these findings, it is suggested that the heightened fold change in miRNA10a contributes to the upregulation of GLUT1 in diabetic patients with OSCC. The interplay between these molecules implies a potential regulatory relationship wherein miRNA10a might influence the expression or regulation of GLUT1 in these pathological conditions, possibly contributing to the observed alterations in glucose metabolism or other relevant pathways in OSCC among diabetic patients.

## Discussion

miRNA10a has an oncogenic role in cancers however its role in OSCC was unknown [10]. miRNA10a proved its oncogenic role by demonstrating OSCC cell proliferation [12,13]. This study unveiled miRNA10a's oncogenic potential by revealing its promotion of OSCC cell proliferation. Additionally, the research established a connection between elevated miRNA10a expression and an increased foldchange in GLUT1, indicating enhanced glucose metabolism.

In OSCC development, there is a documented increase in GLUT1 expression [14,15]. This enhanced GLUT1 presence has previously been linked to cell proliferation in other types of tumors characterized by elevated glucose consumption [16]. The inhibition of GLUT1 has been explored as a potential target for cancer treatment [17]. The current in vitro study illustrated a higher expression of GLUT1 in OSCC compared to both pre-cancerous and healthy control tissues, indicating that increased glucose uptake plays a role in promoting cancer cell growth.

miRNAs are pivotal in regulating cancer progression by modulating glucose levels [12]. Previous research has indicated the promising use of miRNA10a as a screening method for early detection of hepatocellular carcinoma and as a prognostic indicator linked to tumor size [18]. Additionally, studies have reported miRNA10a expression patterns in OSCC [5]. The present in vitro study demonstrated miRNA10a upreguation in OSCC compared to pre-cancerous and healthy control tissue which may be oncogenic via glucose uptake and progression of cancer.

Cancer progression has been demonstrated to be influenced by miRNAs, specifically through their impact on GLUT1 expression [18]. In the current in vitro investigation, a direct correlation was observed between miRNA10a and GLUT1 expression in OSCC. The findings of this research indicate that an increase in miRNA10a foldchange leads to a rise in GLUT1 expression, while elevating GLUT1 expression does not affect miRNA10a expression. Consequently, the results of this study confirm that miRNA10a foldchange promotes glucose uptake by activating GLUT1, promoting cancer growth through enhanced cell proliferation. Previous research has already highlighted the role of miRNAs in regulating GLUT1 and their involvement in cancer growth and progression [5,16].

The present study had a limited sample size. Future investigations should include a larger sample size to further explore the relationship between miRNA10a and GLUT1. Additionally, there is a need for future studies to delve into the molecular pathways connecting miRNA10a and GLUT1.

## Conclusions

This research illuminates the pivotal role played by miRNA10a in the progression of cancer, particularly in its facilitation of proliferation by tightly regulating GLUT1 expression within cancerous tissues, especially under conditions characterized by hyperglycemia. This regulatory mechanism significantly amplifies the transport of glucose within cancer patients, thereby potentially exacerbating the challenges in determining a favorable tumor prognosis. These significant findings underscore and emphasize the immense potential and importance of targeting miRNA10a alongside GLUT1 as viable therapeutic avenues in the comprehensive management of cancer. Intervening the target miRNA and its associated glucose transporter, intervene in the intricate mechanisms underlying cancer progression, potentially offering novel strategies to improve patient outcomes and treatment efficacy.
